# IFISTRATEGY: Spanish National Survey of Invasive Fungal Infection in Hemato-Oncologic Patients

**DOI:** 10.3390/jof9060628

**Published:** 2023-05-30

**Authors:** Carlos Vallejo, Isidro Jarque, Jesus Fortun, Araceli Casado, Javier Peman

**Affiliations:** 1 Hematology Department, Clinic University Hospital of Santiago de Compostela (CHUS), 15706 Santiago de Compostela, Spain; 2 Health Research Institute of Santiago de Compostela (IDIS), 15706 Santiago de Compostela, Spain; 3 Hematology Department, Hospital La Fe, 46026 Valencia, Spain; 4 Centro de Investigación Biomédica en Red de Cáncer (CIBERONC), Instituto Carlos III, 28029 Madrid, Spain; 5 Infectious Diseases Department, Instituto Ramón y Cajal de Investigación Sanitaria IRYCIS, 28034 Madrid, Spain; 6 Investigación Biomédica en Red de Enfermedades Infecciosas (CIBERINFEC), 28805 Madrid, Spain; 7 Pharmacoeconomics and Outcomes Research Iberia (PORIB), 28224 Madrid, Spain; 8 Microbiology Department, Hospital La Fe de Valencia, 46026 Valencia, Spain; 9 Grupo de Investigación Infección Grave, Instituto de Investigación Sanitaria La Fe, 46026 Valencia, Spain

**Keywords:** invasive fungal infection, *Aspergillus fumigatus*, antifungal therapy, azole-resistant *Aspergillus*, emerging molds, new targeted therapies

## Abstract

Recent advances in the treatment of hematologic malignancies have improved the overall survival rate, but the number of patients at risk of developing an invasive fungal infection (IFI) has increased. Invasive infections caused by non-*Candida albicans* species, non-*Aspergillus* molds, and azole-resistant *Aspergillus fumigatus* have been increasingly reported in recent years. We developed a cross-sectional multicenter survey which involved a total of 55 hematologists and infectious disease specialists from a total of 31 Spanish hospitals, to determine the most frequent strategies used for the management of IFIs. Data collection was undertaken through an online survey which took place in 2022. Regarding key strategies, experts usually prefer early treatment for persistent febrile neutropenia, switching to another broad-spectrum antifungal family if azole-resistant *Aspergillus* is suspected, broad-spectrum azoles and echinocandins as prophylactic treatment in patients receiving midostaurin or venetoclax, and liposomal amphotericin B for breakthrough IFIs after prophylaxis with echinocandins in patients receiving new targeted therapies. For antifungals failing to reach adequate levels during the first days and suspected invasive aspergillosis, the most appropriate strategy would be to associate an antifungal from another family.

## 1. Introduction

The emergence of new treatments, such as targeted drugs, for patients diagnosed with hematologic malignancies, has led to a significant reduction in morbidity and mortality among these patients [1], and to a great improvement in their quality of life [2,3]. 

**Figure 1:**
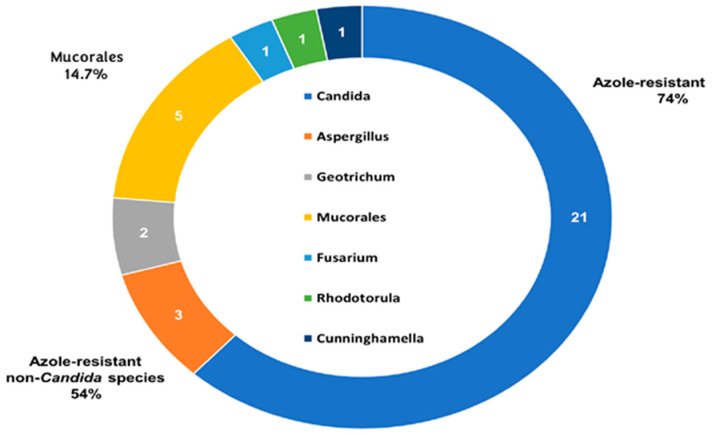
Epidemiology of proven breakthrough invasive fungal infections (IFI) in Spanish patients with hematologic malignancies (adapted from García-Vidal et al. [10]).

However, there has been an increase in the number of possible invasive fungal infection (IFI) hosts, partly due to targeted therapy. For example, cases of invasive aspergillosis (IA) have been described in the first months after starting treatment with ibrutinib, a drug targeting Bruton’s tyrosine kinase (BTK), especially when it is combined with immunosuppressive drugs, such as corticosteroids [4,5]. Moreover, in the past decades, there has been a steady increase of infections produced by non-*Candida albicans* species and non-*Aspergillus* molds, which could be directly related to the extended use of an azole-based antifungal prophylaxis [6] (Figure 1). Finally, there has been a rising concern regarding azole-resistant *Aspergillus* spp. in hematologic patients, which is associated with a higher mortality rate [7]. Prolonged antifungal therapy and the use of triazole pesticides could be responsible for the selection of azole-resistant *Aspergillus fumigatus* and cryptic species [6].

Hematologists and infectious diseases (ID) specialists are well aware of the burden of IFIs, as they often have to deal with these complications in their daily medical practice [8,9,10]. 

We have performed this study with the aim of determining the most frequent strategies used for the management of IFIs in patients with hematologic malignancies. 

## 2. Materials and Methods

Cross-sectional multicenter national survey.

Data collection was accomplished through an online survey which took place between 13 January and 8 February 2022. The survey questionnaire consisted of 12 items. Each question had 4 closed answers. In most cases, it was possible to choose more than one answer. The complete questionnaire and answers provided are shown in Table 1.

## 3. Results

The survey involved a total of 55 hematologists and ID specialists with experience in treating IFIs, from a total of 31 Spanish hospitals. Most of the participating experts were hematologists (64%), while the remaining 36% were ID specialists. The majority of Spanish regions participated on the survey (13 out of 17 regions), which allowed for an almost nationwide assessment. Most of the surveyed experts (78%) were responsible for treating adult patients, compared to 22% who were pediatricians. The mean number of years of practice was 20.5 ± 9.8 years.

1. The latest epidemiological studies on resistance to *Aspergillus* which took place in Spain imply:

Answers provided by the coordinators: (a) We are facing an increase in the resistance of *Aspergillus* spp. to azoles; (b) The percentages are still too low to consider changing the diagnosis/treatment strategy; (c) The need to conduct *Aspergillus* resistance studies on a routine basis; and (d) The possible coexistence of mixed infection (resistant/susceptible *Aspergillus*) does not worry me. 

Comment: Several studies have addressed the trend of *Aspergillus* spp. resistance to antifungal drugs in Spain. Unfortunately, none of these studies have specifically addressed this trend in patients diagnosed with hematologic malignancies. One of the first was a retrospective study which included 362 *A. fumigatus* complex isolates from 150 patients admitted to a large Spanish tertiary hospital in Madrid from 1999 to 2011, and who had been diagnosed with a proven IA, probable IA, or aspergilloma [11]. Most samples were collected from the lower respiratory tract (86,7%). The majority of patients were infected by a single species (*A. fumigatus* (5.3%) and *A. lentulus* (1.3%)). The overall rates of azole resistance of *A. fumigatus* complex and *A. fumigatus* to one or more azoles were 4.2% and 1.8%, respectively. The authors concluded that the rate of azole resistance was very low in *A. fumigatus* strains and that the number of cryptic species isolated was also low. Nonetheless, the authors recommended that a study of several isolates per patient should be performed in order to determine the presence of cases of coinfection by cryptic species, since most cases of coinfection were diagnosed in 2009 or later [11]. Interestingly, a more recent study conducted by the same authors, which involved 725 patients from 29 Spanish hospitals, and which included 847 isolates collected from clinical samples received in the laboratory between 15 February to 14 May 2019, reported an unexpectedly high rate of isolates resistant to at least one azole drug (7.4%) [12]. Resistance was more commonly found in cryptic species than in *A. fumigatus sensu stricto* (95% vs. 5.5%, respectively) [12]. The FILPOP2 Study, a Spanish multicenter epidemiological study, used itraconazole-supplemented plates to screen for azole resistance in 10 tertiary hospitals. A total of 493 isolates were included. The authors observed that although the level of azole resistance in *A. fumigatus* remained low (only 3 out of 260 [1.2%] isolates were resistant to azoles), cryptic species represented 11.5% of the isolates and had a broader but overall higher range of antifungal resistance (in some cases, only amphotericin B showed some activity) [13]. The authors recommended screening for antifungal resistance and performing antifungal susceptibility testing for all isolates collected from sterile sites [13], since antifungal resistance is one of the major causes for mortality in IFIs. A Spanish nationwide antifungal resistance surveillance program, which could determine antifungal resistance and its mechanisms, could help guide the diagnosis and treatment of infections produced by *Aspergillus* spp. resistant to antifungal drugs [14]. 

Results of the survey: For 63.6% of participants, there had been an increase in the resistance of *Aspergillus* spp. to azoles. The possible coexistence of a mixed infection (resistant/susceptible *Aspergillus*) was a concern for 100% of experts. Although 43.6% acknowledged that the percentages were still too low to consider changing the diagnosis/treatment strategy, 41.8% of participants supported the need to conduct *Aspergillus* resistance studies on a routine basis.

2. *Aspergillus* resistance to azoles in hemato-oncological patients is fundamentally:

Answers provided by the coordinators: (a) Of clinical origin, due to the routine use of prophylaxis; (b) Of environmental origin, due to the use of triazole compounds in agriculture; (c) There does not seem to be resistant *Aspergillus* infections in hemato-oncological patients; (d) I would not know how to say it, I lack information.

Comment: The emergence of azole-resistant *A. fumigatus* in high-risk patients such as HSCT recipients was first demonstrated in the Netherlands. Since then, azole-resistant *A. fumigatus* has been increasingly observed worldwide and can seriously compromise the treatment success of IA. A study performed between December 2009 and January 2011 in the Netherlands, which screened 1315 *A. fumigatus* isolates from 921 patients reported a new mediated resistance mechanism (TR46/Y121F/T289A) in 21 azole-resistant isolates from 15 patients in 6 hospitals [15]. Treatment failed in five of eight patients who presented with IA due to TR46/Y121F/T289A and received primary therapy with voriconazole. The study also recovered 140 azole-resistant *A. fumigatus* colonies from 21 locations at 9 different sites. Isolates harboring TR46/Y121F/T289A were not only recovered from epidemiologically unrelated patients, most of whom were azole naive, but were also recovered from environmental samples, suggesting an environmental route of resistance selection [15]. The authors concluded that exposure of *A. fumigatus* to azole fungicides might have facilitated the emergence of new resistance mechanisms, thus compromising the use of azoles in the management of *Aspergillus*-related diseases [15]. Moreover, a previous study also performed in the Netherlands determined that a significant number of patients with an azole-resistant *A. fumigatus* isolate had no history of previous azole exposure during the 3 months before culturing the isolate [16]. Nonetheless, a German study performed in two hematology departments between 2012 and 2013 identified eight patients with an azole-resistant IFI [17]. Although the use of azoles in a clinical setting does not mean that resistance is mediated by azole exposure, five out of eight patients had received prophylactic triazoles [17]. In conclusion, it appears that azole-resistant *Aspergillus* is both clinical and environmental driven.

Results of the survey: For 75% of participants, azole-resistant *Aspergillus* is mainly of clinical origin, due to the routine use of prophylaxis. For 53%, the selection of azole-resistant *Aspergillus is* through a fungicide-driven route.

3. When do you consider it most likely that you will find yourself facing a case of secondary resistance to a broad-spectrum antifungal?

Answers provided by the coordinators: (a) Patient who after a period of improvement presents clinical worsening attributed to his fungal infection; (b) Patient who does not respond to early antifungal treatment administered for 10 days; (c) Patient on antifungal prophylaxis who debuts with symptoms that do not respond to broad-spectrum antibiotics; (d) The probability of secondary resistances is very low.

Comment: Clinical worsening after initial improvement is very subjective and can be observed during recovery from neutropenia (immune reconstitution syndrome). As such, clinicians should not depend solely on the clinical assessment in order to reach a diagnosis. Despite the fact that in recent years there have been important advances in the prevention, diagnosis, and treatment of IFI in patients with hematologic malignancies, the morbidity and mortality associated with these infections is still significant. As previously mentioned, one of the most important prognostic factors that determines the patients’ outcome is mold resistance to antifungal drugs. Resistance can be primary (intrinsic) or secondary (acquired) [18]. Primary resistance is found naturally among molds that have not been exposed to an antifungal drug, while secondary resistance develops among previously susceptible strains after being exposed to an antifungal drug and is usually dependent on altered gene expression [18]. The second scenario could explain cases of IFI produced by strains of *A. fumigatus* or non-*fumigatus Aspergillus* species that have acquired azole resistance or tolerance during prolonged azole exposure in the environment or within the patient. Nonetheless, it must be remarked that secondary resistance with *Candida* spp. is much more common when compared to secondary resistance to molds (which appears to be more unusual). Experts also recommend differentiating between microbiological resistance and clinical resistance [19]. The first would refer to a confirmed non-susceptibility of the mold to an antifungal drug through in vitro susceptibility testing, whereas the latter would be defined as a failure to eradicate an IFI despite the administration of an antifungal agent with in vitro activity against the mold [19]. Clinical failure can be caused by a combination of factors related to the patient, the antifungal drug, and the mold itself. Factors related to patients are fundamentally the net state of immunosuppression (e.g., IA in patients with prolonged severe neutropenia due to marrow failure) [19]. Factors associated with the antifungal drug are mainly its pharmacokinetic/pharmacodynamic characteristics (drug–drug interactions, difficulty in absorption or differences in the drugs’ metabolisms which can lead to the emergence of toxicities), the infection site (e.g., penetration in the central nervous system [SNC] can dramatically vary between antifungal families), and the length of treatment (lack of compliance or a suboptimal length of treatment can often lead to clinical failure) [19]. Factors associated with the mold itself are its burden (a higher inoculum of the mold is usually associated with clinical failure), its intrinsic virulence, and the ability to form biofilms [19]. 

Result of the survey: According to the experts, a clinician could be facing a case of secondary resistance to a broad-spectrum antifungal whenever a patient presents clinical worsening attributed to their fungal infection after a period of improvement (31.5%), in patients who do not respond to early antifungal treatment administered for 10 days (29.6%), and in patients on antifungal prophylaxis who begin with symptoms that do not respond to broad-spectrum antibiotics (27.8%).

4. In the event of suspected resistance in a patient receiving treatment for aspergillosis, what strategy would you carry out?

Answers provided by the coordinators: (a) Change of antifungal family to another broad-spectrum; (b) Combined treatment with two new antifungals from different families; (c) Association of another broad-spectrum antifungal from a different family; (d) Increase the dose of the antifungal in use, if possible.

Comment: There is limited clinical data available that can help decide on the best treatment option in the case of an azole-resistant IA. Nevertheless, experts agree that since azole-resistant is associated with a high mortality rate, a change of therapy should be made as soon as resistance is confirmed, irrespective of the duration and dose of the prescribed azole drug [20]. The Study Group of Fungal Infections (GEMICOMED, by its acronym in Spanish) from the Spanish Society of Infectious Diseases and Clinical Microbiology (SEIMC, by its acronym in Spanish) guidelines recommend amphotericin B (AIII) or a combination of voriconazole with an echinocandin (CIII) in case of IA caused by cryptic species or by isolates resistant to voriconazole (MIC > 2 mg/L) [21]. The choice on the best treatment option will depend on the in vitro susceptibility data of the isolate, site of infection, and patient characteristics [20,21]. 

**Figure 2:**
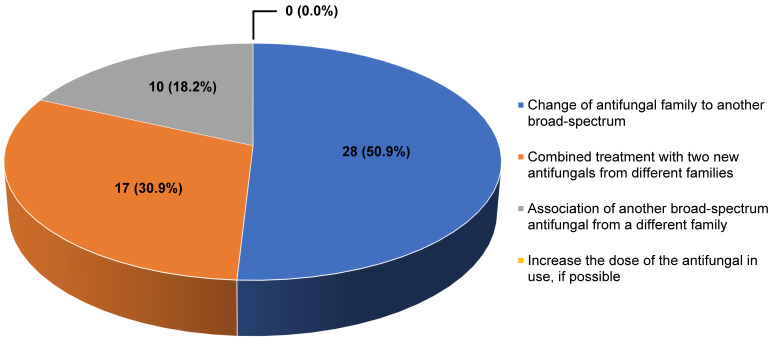
Answers provided by the experts to the question “In the event of suspected resistance in a patient receiving treatment for aspergillosis, what strategy would you carry out?”.

Result of the survey: 50.9% of the participants would change to a broad-spectrum antifungal drug from a different family while 30.9% would combine two new antifungals drugs from different families. None would increase the dose of the antifungal drug (Figure 2).

5. The IDSA and ESCMID Guidelines recommend modifying the therapeutic strategy when the percentage of resistance of *A. fumigatus* against an azole is ≥10%. In your opinion, this may imply…

Answers provided by the coordinators: (a) A change in the choice of early treatment; (b) A change in the choice of prophylactic treatment; (c) In my area there are no *A. fumigatus* that show resistance to azoles; and (d) Without a previous in vitro susceptibility study, I would not worry.

Comment: The increase of the rates of *A. fumigatus* to azoles is a clinical concern, as it is usually associated with a poor clinical outcome. As such, recent published guidelines have thoroughly addressed the first-line treatment in the case of a possible azole-resistant IA. The GEMICOMED-SEIMC guidelines recommend that in areas with a high rate of azole resistance >10%, azole monotherapy should be avoided as an empirical primary treatment, especially in the case of severe cases of IA (BIII) [21]. In that case, amphotericin B (AIII) or a combination of voriconazole with an echinocandin (CIII) is recommended. The 2017 European Society for Clinical Microbiology and Infectious Diseases, the European Confederation of Medical Mycology and the European Respiratory Society (ESCMID-ECMM-ERS) joint clinical guidelines recommend the use of a first-line therapy with liposomal amphotericin B (L-AMB) (BIII) or voriconazole plus echinocandin (BIII) whenever azole resistance rates are >10% [22]. 

Results of the survey: A percentage of 87% indicated that a change should be made in the choice of early treatment.

6. In the face of persistent febrile neutropenia (5 days), what would you do regarding antifungal treatment?

Answers provided by the coordinators: (a) I would initiate it in the presence of IFI-specific pulmonary infiltrate; (b) I would initiate it if positivity of any biomarker (GM, BDG…) regardless of the result of the imaging test (CT); (c) I would initiate it in the presence of nonspecific or specific IFI infiltrate; and (d) I would initiate it in the absence of a pulmonary infiltrate.

Comment: IA can have a dismal outcome in hematologic patients and delayed treatment is associated with a higher mortality rate [23]. It cannot be overstated that, classically, the most prevalent but unspecific sign of invasive pulmonary aspergillosis (IPA) is persistent fever despite treatment with broad-spectrum antibiotics [24], and that when persistent fever exists but the chest CT scan is considered normal, the clinician should consider other foci of IFI apart from the lungs [23]. The ESCMID-ECMM-ERS joint clinical guidelines suggest that patients with prolonged neutropenia (<500/µL for ≥96 h), receiving induction or remission chemotherapy for acute leukemia or myelodysplastic syndrome or conditioning chemotherapy for HSCT, and with fever (>38 °C) despite parenteral broad-spectrum antibacterial therapy for more than 96 h, would be candidates for receiving empirical antifungal therapy, in what would be considered a “fever-driven” approach [22]. The GEMICOMED-SEIMC guidelines highlight that in the case of high-risk patients already receiving anti-mold prophylaxis, empirical antifungal treatment could be waived in spite of fever that does not respond to antibiotic therapy (AII) [21]. Nowadays, very few clinicians wait for IFI-specific pulmonary infiltrates in order to start antifungal treatment and an empirical antifungal approach is still widely used in high-risk patients with neutropenia when a prompt diagnostic work-up cannot be performed [25]. Finally, a recent open-label phase 3, randomized, parallel, multicenter, strategy trial which compared empirical versus pre-emptive antifungal strategies in high-risk neutropenic patients on fluconazole prophylaxis concluded that the pre-emptive antifungal strategy was safe in these patients [26]. The authors also concluded that a pre-emptive strategy would be particularly useful in centers that do not use mold-active azole prophylaxis or in patients who cannot be on mold-active azole prophylaxis [26].

**Figure 3:**
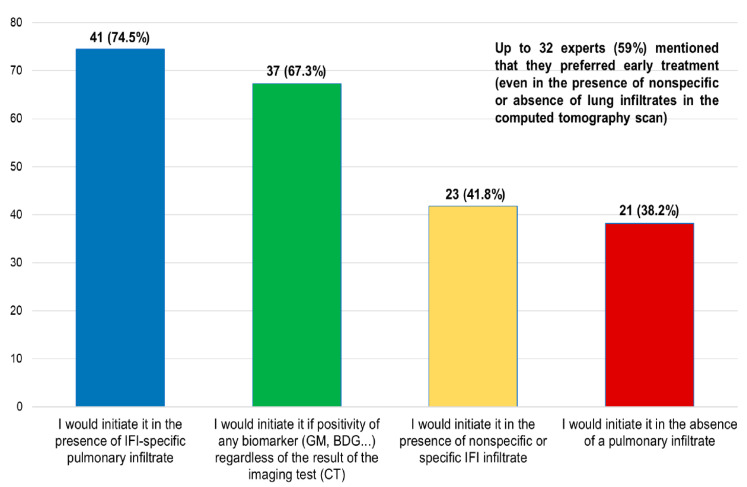
Answers given to the question ”In the face of persistent febrile neutropenia (5 days), what would you do regarding antifungal treatment?”.

Results of the survey: 59% of the surveyed participants indicated that they would perform early treatment (even in presence of nonspecific or absence of lung infiltrates in the CT) (Figure 3). The high rate of physicians who prefer empirical treatment can be explained by the high mortality rate associated with IFIs in these patients, and by the delay in obtaining the results of serum markers (e.g., galactomannan) and imaging tools. Although available in most centers, there can be a considerable delay between extracting the sample, performing the exams, and delivering the results to the physician; this justifies the more frequent use of empirical treatments.

7. Regarding breakthrough IFIs in Spain

Answers provided by the coordinators: (a) An increase in the incidence of Mucorales has been observed; (b) Proven ones are often resistant to previously administered antifungals; (c) They are associated with a change in epidemiology; and (d) Mortality from IFIs has decreased.

Comment: A prospective, observational, and multicenter study with breakthrough IFI in hematologic patients was conducted in 13 Spanish university hospitals for 36 months [27]. Patients diagnosed with acute leukemia and HSCT were included. Antifungal susceptibility was tested in the Spanish National Center for Microbiology. A total of 121 episodes were included, with 41 cases of proven IFI (principally, 20 cases of non-*Candida albicans*, 7 cases of Mucorales, 3 cases of *Aspergillus*, and 2 cases of *Fusarium solani*). Overall, in 30.6% of patients there was IFI progression, and the mortality rate was 47.1%. The authors concluded that breakthrough IFIs were fundamentally caused by rare molds (Mucorales or *Fusarium* spp.), non-*fumigatus Aspergillus* and non-*Candida albicans* species, which were resistant to the prior antifungal drug administered, and had a direct impact on the prognosis with an extremely high 100-day mortality rate. An Austrian single-center retrospective study which evaluated 99 hematologic patients who had undergone 202 courses of primary antifungal prophylaxis with posaconazole due to high risk of IFI reported that all breakthrough IFIs had been caused exclusively by non-*Aspergillus* species, and particularly by Mucorales [28]. Finally, a retrospective analysis which compared 24 microbiologically documented breakthrough IFIs that occurred during posaconazole or voriconazole prophylaxis with 66 non-breakthrough IFIs showed a shift towards non-*Aspergillus* molds and a significant increase of rare multidrug-resistant molds [29]. Compared to the non-breakthrough IFIs, the proportion of infections caused by Mucorales increased from 15% to 31%, while the rate of infections produced by *Aspergillus* spp. decreased from 56% to 24% (moreover, 57% were caused by non-*Aspergillus fumigatus*) [29]. The results of these studies confirm that in the past decades the epidemiology of IFIs in hematological patients has been changing, with a decrease of *Candida albicans* infections, accompanied by a progressive increase of infections produced by non-albicans *Candida* species, filamentous molds, and/or multidrug-resistant molds [30]. It is expected that this trend will continue due to the increased use of mold-active antifungals and the increase in the survival rate of patients diagnosed with hematologic malignancies [30]. Diagnostic and therapeutic decisions in a patient with a breakthrough IFI, especially if the patient is receiving prophylaxis with posaconazole, is still challenging [31]. L-AMB, as a first-line empirical therapy for breakthrough IFIs, would be the recommended treatment in patients who had received an azole-based prophylactic treatment [10,30,31,32]. In the future, better clinical data and individualized decisions are the best means to improve the outcomes of patients who develop breakthrough IFIs while receiving mold-active antifungal drugs [31]. 

Results of the survey: 65% of the experts indicated an increase in the incidence of Mucorales, with 65% mentioning that proven IFIs were usually resistant to previously administered antifungals. Moreover, 62% mentioned that breakthrough IFIs are associated with a change in epidemiology. In addition, 16.4% considered that IFI-associated mortality has decreased.

8. Regarding the monitoring of serum levels of azoles (e.g., voriconazole), what is the situation in your hospital?

Answers provided by the coordinators: (a) We obtain the results between 1 and 3 days from the taking of the sample; (b) We obtain the results in less than 24 h from taking the sample; (c) We obtain the results between 4–5 days from the taking of the sample; and (d) Normally we need more than five days, or we do not have them.

Comment: The GEMICOMED-SEIMC and ESCMID-ECMM-ERS joint clinical guidelines recommend therapeutic drug monitoring (TDM) of azoles (AII) [21,22]. The first sample should be obtained once a steady state has been reached (usually from 3 to 7 days depending on the antifungal drug) (AI), while the following samples should be obtained at least once a week after dose stability is achieved (CIII) [21]. The serum trough levels usually depend on the azole drug prescribed, whether the drug is prescribed as prophylactic therapy or as treatment for an IA, and the severity of the infection (e.g., disseminated disease, CNS involvement, or higher minimum inhibitory concentration) [22]. When the trough concentration is not reached or is exceeded, the drug dosage should be increased or decreased, respectively (AIII) [21]. TDM would be especially useful for monitoring itraconazole, voriconazole, posaconazole, and flucytosine, as these agents demonstrate a well-defined exposure–response relationship and an unpredictable pharmacokinetic profile or a narrow therapeutic index [33]. However, TDM is not routinely recommended for amphotericin B, fluconazole, isavuconazole, and echinocandins [33], although isavuconazol is frequently used in clinical situations in which TDM is recommended [34]. In conclusion, TDM is a sensible way to identify patients receiving azole therapy and who are at risk of treatment failure or drug-induced toxicity (e.g., patients with hepatic or renal impairment, or who are at high-risk of developing drug–drug interactions). TDM can be extremely useful for adjusting the antifungal dosing, thus improving the patients’ outcome.

Results of the survey: 22% of the participants mentioned that they are able to obtain the results of serum levels of azoles in less than 24 h from taking the sample, whereas 46% and 17% obtain the results between 1–3 days and between 4–5 days from taking the sample, respectively. Nonetheless, approximately 15% of the experts mentioned that they normally need more than 5 days to obtain them or simply do not have them available.

9. Some antifungals do not reach levels during the first days of their administration. In this situation, in case of IA suspicion, what strategy do you think would be the most appropriate?

Answers provided by the coordinators: (a) Associate an antifungal from another family and perform levels before returning to monotherapy; (b) Check that the patient is not at risk of low levels due to interactions (e.g., dexamethasone) and maintain monotherapy; (c) None of the options; and (d) Associate an antifungal from another family and wait for the patient’s clinical improvement.

Comment: According to the guidelines, voriconazole is the recommended primary treatment for IA in patients with hematologic malignancy [21,22,35]. Voriconazole’s pharmacokinetics and pharmacodynamics have been well established, and several studies have been published concerning its absorption, distribution, metabolism, and elimination. It must be highlighted that voriconazole shows time-dependent slow fungicidal activity and a short post-antifungal effect against *Aspergillus* species [36]. The drug is rapidly absorbed within 2 h after oral administration and its oral bioavailability is over 90% (voriconazole should be administered 1 h before or after meals, since food lowers voriconazole’s bioavailability and delays absorption) [36]. Voriconazole shows nonlinear pharmacokinetics due to its capacity-limited elimination, and its pharmacokinetics are dependent upon the administered dose [36]. As such, the steady-state plasma concentrations are reached approximately 5 days after both intravenous and oral administration [36]. It must also be strengthened that several case reports and studies have highlighted that voriconazole concentrations could be subtherapeutic in patients receiving dexamethasone, thus increasing the probability of treatment failure [37,38,39]. Dexamethasone is a strong inducer of CYP3A4 and a moderate inducer of CYP2C9 and CYP2C19. By inducing CYP3A4 and CYP2C19 enzymes, dexamethasone could lead to rapid metabolism of voriconazole and subtherapeutic serum concentrations. In one case report, due to persistent fever despite therapy with voriconazole, the impossibility of obtaining therapeutic concentrations, and the suspicion of a severe fungal infection, voriconazole was replaced by L-AMB. The authors considered that dexamethasone was most likely responsible for the impossibility of obtaining therapeutic concentrations of voriconazole [37]. Combination therapy could also optimize the treatment in these cases. A retrospective single-center cohort study, which included 550 consecutive adult allogeneic HSCT recipients from 1 January 2010 to 1 January 2020, described that antifungal combination therapy was common, with almost one third of patients receiving at least 7 days of antifungal combination therapy during the first month of treatment [40]. Remarkably, combination treatment was used to avoid low azole serum drug levels in 9 (19%) out of 47 courses. The authors explained that due to the time elapsed before reaching a steady state for most broad-spectrum azoles, the problems related to absorption in high-risk patients with mucositis or gastrointestinal graft-versus-host disease (GvHD), the multiple drug interactions, which affect drug absorption and metabolism, and the severity of IA in patients with hematologic malignancies and allogeneic HSCT recipients, combination treatment could be prescribed as a bridge until therapeutic azole concentrations are reached in these high-risk fragile patients [40]. 

**Figure 4:**
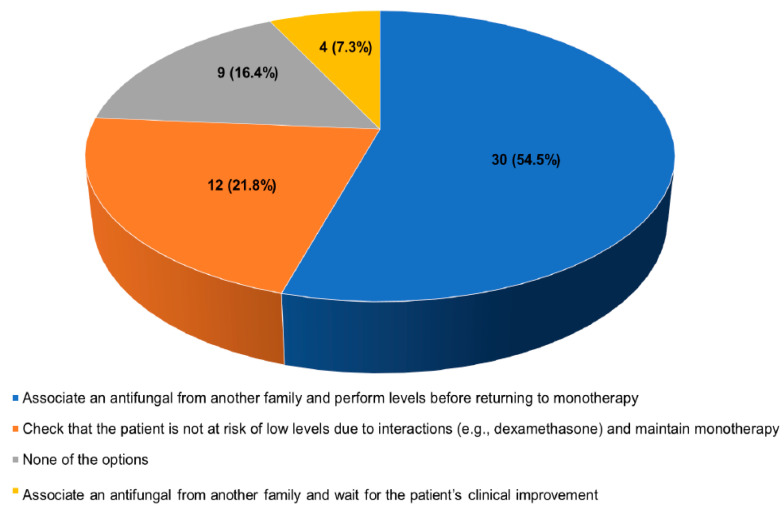
Answers provided by the experts to the question “Some antifungals do not reach levels during the first days of their administration. In this situation, in case of IA suspicion, what strategy do you think would be the most appropriate?”.

Results of the survey: The most appropriate strategy for 62% of the surveyed experts would be to associate antifungal drugs from different families; nonetheless, 22% would check that the patient was not at risk of low levels due to interactions (e.g., dexamethasone) and maintain monotherapy (Figure 4).

10. Some of the newer targeted therapy drugs have interactions with antifungals. In this context, if indicated, what type of prophylaxis would you administer in a patient receiving midostaurin or venotoclax?

Answers provided by the coordinators: (a) Extended spectrum azoles; (b) Echinocandin; (c) Others; and (d) Fluconazole.

Comment: Midostaurin is a multikinase inhibitor approved by the European Medicines Agency (EMA) and the Spanish Agency of Medicines and Medical Devices (AEMPS, by its acronym in Spanish) for adult patients: (1) with a newly diagnosed acute myeloid leukemia (AML) who are FLT3-mutation positive, in combination with standard daunorubicin and cytarabine induction and high-dose cytarabine consolidation chemotherapy; (2) as a single-agent maintenance therapy for adult patients with a complete response; and (3) as monotherapy for the treatment of adult patients with aggressive systemic mastocytosis, systemic mastocytosis with associated hematological neoplasm, or mast cell leukemia [41]. Venetoclax is a potent, selective inhibitor of BCL2, approved by the EMA and the AEMPS: (1) in combination with azacytidine or decitabine, for the treatment of newly diagnosed AML in adults who have comorbidities that preclude the use of intensive approaches and (2) for treatment of chronic lymphocytic leukemia (CLL) in combination with obinutuzumab or rituximab, or as monotherapy [42]. Both midostaurin and venetoclax are primarily metabolized by CYP3A4. Co-administration with strong CYP3A4 inhibitors, such azoles, could increase drug exposure and could induce toxicity and drug-induced side effects [43]. A study which included 12 patients receiving different doses of venetoclax with posaconazole (400 mg venetoclax monotherapy after ramp-up, 50 mg venetoclax with 300 mg posaconazole, or 100 mg venetoclax with 300 mg) and which collected blood samples before dosing and up to 24 h after the venetoclax dose on days 20 and 28, reported that when adjusted for different doses and nonlinearity, posaconazole had venetoclax Cmax and AUC0–24 increased by 7.1- and 8.8-fold, respectively [44]. Importantly, both the 50- and 100-mg venetoclax doses administered with posaconazole were well tolerated, and the authors concluded that posaconazole could be used for antifungal prophylaxis in patients with AML receiving venetoclax after reducing the venetoclax dose by at least 75% [44]. A retrospective cohort study which included 277 adult patients with newly diagnosed AML undergoing treatment with a high- or low-intensity with venetoclax-containing therapy and active triazoles as primary antifungal prophylaxis (51% posaconazole, 30% voriconazole, 19% isavuconazole) described that 38 (14%) of patients had to discontinued prophylaxis due to toxicity, primarily hepatotoxicity [45]. Discontinuation due to hepatotoxicity was similar among the three azoles [45]. Finally, a retrospective and observational study which included four patients receiving posaconazole and midostaurin described that the plasma concentrations of midostaurin were eight times higher than in patients not receiving posaconazole [46]. The expert consensus recommendation from the European Hematology Association considers that, when midostaurin or venetoclax are administered to patients with a high risk of developing IFIs, a prophylactic therapy with triazoles, or specifically posaconazole, should be preferably used [47]. Close monitoring with dose reduction of the new targeted hematological drugs, whenever necessary, and a close follow-up for adverse events should be performed [47,48,49]. It is important to remark that these recommendations could be revised in the future as more data becomes available, and that L-AMB or echinocandins, which do not inhibit CYP enzymes, could also be considered as an alternative to triazoles in patients requiring antifungal prophylactic therapy [48,49]. It must be remarked that, although echinocandins show poor CNS penetration, lack coverage against some Mucorales and *Fusarium* species [49], and are fungistatic against *Aspergillus*, they have a lower rate of drug–drug interactions, and a lower rate of drug-related adverse events when compared to azoles; this could favor the use of echinocandins instead of azoles in patients receiving targeted therapy [50]. 

Results of the survey: Most clinicians would administer extended spectrum azoles (40.0%) or echinocandin drugs (38.2%); approximately 21.8% of the experts surveyed would use other antifungal drugs than azoles or echinocandins, such as nebulized antifungals; and none would use fluconazole. It must be remarked that all clinicians who participated in this survey mentioned that they have used venetoclax in their daily clinical practice in patients diagnosed with AML, a hematological disease with a high risk for developing an IFI.

11. If echinocandins were used as prophylaxis in a patient receiving midostaurin or venetoclax, in case of suspected breakthrough fungal infection, what treatment would you administer?

Answers provided by the coordinators: (a) Liposomal amphotericin B; (b) Isavuconazole; (c) Voriconazole; and (d) A combined treatment.

Comment: In the case of a patient receiving prophylactic treatment with echinocandins and a suspected breakthrough infection, the GEMICOMED-SEIMC guidelines recommend initiating empirical treatment with an alternative class of antifungal drugs until the diagnosis is established and a response to treatment is documented (B-III) [21]. The ESCMID-ECMM-ERS joint clinical guidelines also recommend a switch to a different class of antifungal drugs than those previously used as prophylactic therapy [22]. As we have already mentioned, midostaurin and venetoclax are primarily metabolized by CYP3A4 and their co-administration with azoles, which are strong CYP3A4 inhibitors, could induce toxicity and drug-induced side effects. There is very limited data on the use of voriconazole as a treatment in patients receiving midostaurin or venetoclax. A patient diagnosed with CLL and who was receiving voriconazole for a CNS IA was concomitantly treated with venetoclax, without drug-induced side effects [51]. Importantly, the patient’s initial dose of venetoclax was 10 mg daily, which was subsequently increased to 50 mg, and ultimately escalated to 100 mg due to the absence of side effects or toxicity. Nevertheless, the patient was under very strict surveillance [51]. Due to the lack of clinical data and the severity of IA in a patient with hematologic malignancy, it appears sensible to explore other alternatives to triazoles in patients who require antifungal treatment [48]. As such, L-AMB could be a suitable solution as an antifungal treatment in patients receiving midostaurin or venetoclax [10,48]. Moreover, if there is a suspected breakthrough IFI while receiving echinocandin prophylactic therapy L-AMB would allow for a broad-spectrum antifungal treatment, while maintaining the antineoplastic therapy due to the lack of drug–drug interactions [10]. Isavuconazole, which appears to be a lesser CYP3A4 inhibitor has a good safety profile and a more easily managed drug–drug interaction with immunosuppressive agents, could also be a possible treatment alternative [52]. 

**Figure 5:**
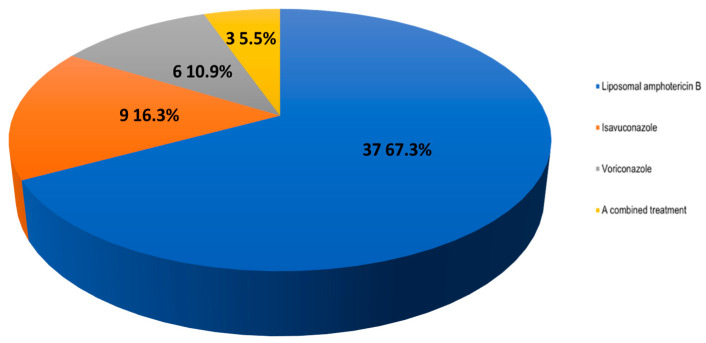
Answers to the question “If echinocandins were used as prophylaxis in a patient receiving midostaurin or venetoclax, in case of suspected breakthrough fungal infection, what treatment would you administer?”.

Results of the survey: 67.3% would recommend L-AMB, 16.4% would recommend isavuconazole, 10.9% would use voriconazole, and 5.5% would prescribe a combined treatment (Figure 5). Interestingly, when comparing the answers provided by pediatricians and by physicians who attend adult patients, there were no significant differences in the use of L-AMB or voriconazole (83.3% vs. 62.8% and 8.3% vs. 11.6%, respectively). Nonetheless, the use of isavuconazole was solely considered by the physicians who attend adult patients (0.0% vs. 20.9%, respectively).

12. Regarding cryptococcosis in the hematological patient

Answers provided by the coordinators: (a) I take it into account, but I have not seen recent cases in the hospital; (b) I usually take it into account and if necessary, I carry out the necessary tests; (c) It is underdiagnosed, it is not usually taken into account; and (d) It is not relevant in the hematological patient.

Comment: *Cryptococcus* spp. is a saprophytic encapsulated yeast, responsible for producing opportunistic infections in immunosuppressed patients. Although hematological patients have an increased risk of developing IFIs by filamentous fungi, infections produced by *Cryptococcus* occur much more rarely [53]. A Brazilian retrospective, single-center cohort study which included consecutive patients with hematologic malignancies or undergoing HSCT who had been diagnosed with proven or probable IFIs between 2009 and 2019, reported that cryptococcosis was the fourth most common IFI (8 out of 94 cases [8.5%]) [54]. All cases were caused by *Cryptococcus neoformans*. Fungemia occurred in six cases, whereas in one case there was involvement of the lungs and in one case involvement of both the lungs and the CNS. The 6-week mortality rate was of 37.5% [55]. A nationwide Finnish retrospective study which included 22 patients who had received antifungal treatment for cryptococcosis from January 2004 to December 2018 reported that of the 12 human immunodeficiency virus (HIV)-negative patients included, 5 (41.7%) had a hematologic malignancy [55]. The authors concluded that, although cryptococcosis was a rare disease in Finland, in their case series the most common immunocompromising disease after HIV was hematologic malignancy [55]. Recently, there has been an increasing number of cases of cryptococcosis in patients receiving ibrutinib, especially in the first months of treatment [6,56]. A study which included 19 cases of patients who developed cryptococcosis while on ibrutinib, reported that disseminated cryptococcosis was frequent (52.6% of cases), with lung and CNS involvement reported in 68.4% and 42.1% of cases, respectively. Five patients eventually died, although in three cases this was due to unrelated cases. Ibrutinib, more than three lines of chemotherapy, and neutropenia were considered as risk factors for developing cryptococcosis [56]. In conclusion, clinicians should be aware of the emerging risk of cryptococcal infections in hematologic patients. A high suspicion rate for the diagnosis and treatment is extremely important, as *Cryptococcus* infections are life threatening, especially in patients with CNS involvement. Diagnosis is usually delayed, which leads to a poor prognosis and long-term neurologic sequelae. Early recognition is essential for an earlier start of the treatment and favors a complete recovery. Further research is mandatory.

Results of the survey: Almost 80% of the experts answered that they take it into account, but have not diagnosed recent cases; 23.6%, mentioned that, if necessary, they would carry out the necessary tests; and interestingly, 5.5% reported that *Cryptococcus* spp. is not relevant in hematological patients.

## 4. Discussion

This study describes the management of patients with hematologic malignancies and HSCT, who are at risk of developing IFIs. Based on these results, we can conclude that most of the experts agree on: (1) if resistance of *Aspergillus* to azoles is suspected, switching to another broad-spectrum antifungal family such as L-AMB would be the best option; (2) early antifungal treatment is the best option in case of persistent febrile neutropenia (even in the presence of nonspecific or absence of lung infiltrate in the CT); (3) for antifungal drugs failing to reach adequate levels during the first days, and if IA is suspected, the most appropriate strategy would be the association of an antifungal of another family; (4) there was no consensus on which prophylaxis (broad-spectrum azoles or echinocandins) should be used in patients receiving new targeted therapies, such as midostaurin and venetoclax; and (5) L-AMB was the preferred option in case of breakthrough IFIs in patients receiving new targeted therapies and prophylactic therapy with echinocandins.

## Figures and Tables

**Table 1:** Questionnaire and answers provided by the 55 experts who participated in the survey.

	Answers ^a^ n (%)
**1. The latest epidemiological studies on resistance to *Aspergillus* carried out in Spain imply…**	
That we are facing an increase in *Aspergillus* resistance to azoles	35 (63.6)
The percentages are still too low to consider changing the diagnosis/treatment strategy	24 (43.6)
The need to conduct *Aspergillus* resistance studies on a routine basis	23 (41.8)
The possible coexistence of mixed infection (resistant/susceptible *Aspergillus*) does not worry me	0 (0.0)
**2. *Aspergillus* resistance to azoles in hemato-oncology patients is fundamentally…**	
Of clinical origin, due to the routine use of prophylaxis	41 (74.5)
Of environmental origin, due to the use of triazole compounds in agriculture	29 (52.7)
There do not seem to be resistant *Aspergillus* infections in hemato-oncological patients	3 (5.5)
I would not know how to say it, I lack information	3 (5.5)
**3. When do you consider it most likely that you will find yourself facing a case of secondary resistance to a broad-spectrum antifungal?**	
Patient who after a period of improvement presents clinical worsening attributed to his fungal infection	17 (31.5)
Patient who does not respond to early antifungal treatment administered for 10 days	16 (29.6)
Patient on antifungal prophylaxis who debuts with symptoms that do not respond to broad-spectrum antibiotics	15 (27.8)
The probability of secondary resistances is very low	6 (11.1)
**4. In the event of suspected resistance in a patient receiving treatment for aspergillosis, what strategy would you carry out?**	
Change of antifungal family to another broad-spectrum	28 (50.9)
Combined treatment with two new antifungals from different families	17 (30.9)
Association of another broad-spectrum antifungal from a different family	10 (18.2)
Increase the dose of the antifungal in use, if possible	0 (0.0)
**5. The IDSA and ESCMID Guidelines recommend modifying the therapeutic strategy when the percentage of resistance of *A. fumigatus* against an azole is ≥10%. In your opinion, this may imply…**	
A change in the choice of early treatment	48 (87.3)
A change in the choice of prophylactic treatment	17 (30.9)
In my area there are no *A. fumigatus* that show resistance to azoles	11 (20.0)
Without a previous in vitro susceptibility study, I would not worry	2 (3.6)
**6. In the face of sustained febrile neutropenia (5 days), what would you do regarding antifungal treatment? ^b^**	
I would initiate it in the presence of IFI-specific pulmonary infiltrate	41 (74.5)
I would initiate it if positivity of any biomarker (GM, BDG…) regardless of the result of the imaging test (computed tomography)	37 (67.3)
I would initiate it in the presence of nonspecific or specific IFI infiltrate	23 (41.8)
I would initiate it in the absence of a pulmonary infiltrate	21 (38.2)
**7. Regarding breakthrough IFIs in Spain…**	
An increase in the incidence of mucorales has been observed	36 (65.5)
Proven ones are often resistant to previously administered antifungals	36 (65.5)
They are associated with a change in epidemiology	34 (61.8)
Mortality from IFIs has decreased	9 (16.4)
**8. Regarding the monitoring of serum levels of azoles (e.g., voriconazole), what is the situation in your hospital?**	
We obtain the results between 1 and 3 days from the taking of the sample	25 (46.3)
We obtain the results in less than 24 h from taking the sample	12 (22.2)
We obtain the results between 4–5 days from the taking of the sample	9 (16.7)
Normally we need more than 5 days, or we do not have them	8 (14.8)
**9. Some antifungals do not reach levels during the first days of their administration. In this situation, in case of IA suspicion, what strategy do you think would be the most appropriate?**	
Associate an antifungal from another family and perform levels before returning to monotherapy	30 (54.5)
Check that the patient is not at risk of low levels due to interactions (e.g., dexamethasone) and maintain monotherapy	12 (21.8)
None of the options	9 (16.4)
Associate an antifungal from another family and wait for the patient’s clinical improvement	4 (7.3)
**10. Some of the newer targeted therapy drugs have interactions with antifungals. In this context, if indicated, what type of prophylaxis would you administer in a patient receiving midostaurin or venotoclax?**	
Extended spectrum azoles	22 (40.0)
Echinocandin	21 (38.2)
Others	12 (21.8)
Fluconazole	0 (0.0)
**11. If echinocandins were used as prophylaxis in a patient receiving midostaurin or venetoclax, in case of suspected breakthrough fungal infection, what treatment would you administer?**	
Liposomal amphotericin B	37 (67.3)
Isavuconazole	9 (16.4)
Voriconazole	6 (10.9)
A combined treatment	3 (5.5)
**12. Regarding cryptococcosis in the hematological patient…**	
I take it into account, but I have not seen recent cases in the hospital	44 (80.0)
I usually take it into account and if necessary, I carry out the necessary tests	13 (23.6)
It is underdiagnosed, it is not usually taken into account	9 (16.4)
It is not relevant in the hematological patient	3 (5.5)

BDG: 1,3-β-d-glucan; GM: galactomannan; IA: invasive aspergillosis; IFI: invasive fungal infections. ^a^ Each question had 4 closed answers, and in most cases it was possible to choose more than one answer. ^b^ Up to 32 experts (59%) mentioned that they preferred early treatment (even in the presence of nonspecific or absence of lung infiltrates in the computed tomography scan).

## Data Availability

Not applicable.
